# Correction: Yoo, J.Y., et al. Gut Microbiota and Immune System Interactions. *Microorganisms* 2020, *8*, 1587

**DOI:** 10.3390/microorganisms8122046

**Published:** 2020-12-21

**Authors:** Ji Youn Yoo, Maureen Groer, Samia Valeria Ozorio Dutra, Anujit Sarkar, Daniel Ian McSkimming

**Affiliations:** 1 College of Nursing, University of South Florida, Tampa, FL 33612, USA; mgroer@usf.edu (M.G.); anujit@usf.edu (A.S.); 2 College of Nursing, University of Tennessee-Knoxville, Knoxville, TN 37916, USA; sozoriod@utk.edu; 3 College of Public Health, University of South Florida, Tampa, FL 33612, USA; 4 Morsani College of Medicine, University of South Florida, Tampa, FL 33612, USA; dmcskimming@usf.edu

Corrections have been made to “Gut Microbiota and Immune System Interactions” [1], a review article published in *Microorganisms*, to remove text found in previously published work. While phrasing was adjusted throughout, the most extensive changes can be found in Sections 2.1 and 3.1.

Within days of publication, the authors became aware of some unintentional language replication. Albeit cited, several references were not properly paraphrased or quoted from the sources. After contacting the journal and offering to retract the article, we conducted an extensive review of the manuscript and made necessary adjustments. Details of modifications were provided to the journal for review, and corrections were deemed sufficient in lieu of retraction. Only stylistic editorial changes were made and thus there is no alteration in content from the originally accepted manuscript.

We would like to thank *Microorganisms*’ Editorial Board for allowing us to make corrections and offer our sincerest apology to the scientific community.
